# Obstetrics in the Sandwich Islands

**Published:** 1872-10

**Authors:** Chas. H. Wetmore


### ART. IV.—*Obstetrics in the Sandwich Islands.* By CHAS. H. WETMORE, M. D.

In order to know how to prize any branch of science properly we need to look backward and note its origin and allow the mind's eye to follow it on in its progress of development from its native to its real, or approximate, zenith. Civilized and enlightened nations are apt to look upon their attainment in both the sciences and the arts as matters, which have been handed down to them from former generations very much as legacies or titles to extensive and valuable landed estates descend from a parent to his child or from the nobleman to his oldest heir, forgetful of the diligent students' labors and efforts, both in and out of his studio to cause his department of science to occupy the position due to it among the educated and refined.

Let me invite my readers to accompany me in imagination to a group of Islands raised up by volcanic agencies in the great Pacific Ocean two thousand miles west of San Francisco, forming an important "way-station" on that new Ocean high-way of nations. Rude and barbarous were the people only a half century ago, and a half century earlier than that period the existence of that "pin-head kingdom" was unknown to our most skilful navigators. The inhabitants of those Sandwich Islands have been "civilized and christianised" while many and perhaps most of them retain not a little of their crude, ancient and sometimes superstitious ideas and practices; it requires more than a generation and a half to divest any nation of such habits of life and thought; our own national experience proves this assertion.

In this article your attention is particularly called to their obstetrical practice as I have witnessed it during a residence of twenty-two years on Hawaii. When the period of utero gestation is nearly completed many of them drink freely of a mucilage prepared from the inner bark of the "Haw" or Hibiscus tree much as some American women drank of a nostrum intended to prepare the system to do its duty properly at such a period.—When labor is fairly commencing the patient assumes a sitting posture upon a hard pillow or stone, with her husband or some other intimate *male* or female friend, resting upon his or her knees behind her, whose duty it is to grasp her above the abdomen in such a way that he or she can press down with considerable force upon the uterus and its contents never relaxing this grasp to allow the fœtus to recede; the accoucher's position is in front. He, (or *usually she,*) has but little to do but receive the child; if the case is at all tedious or prolonged they imagine it a "cross birth," or that the child is dead, or something else, *never certain* what presentation exists until the head or some other part shows itself externally. They seldom allow the cord to be tied or cut until the after-birth comes away; to effect its speedy removal, which they regard as very desirable, and necessary, they place the mother in a semi-erect posture with the pelvis thrown backward and the knees partly flexed, the midwife at the same time supporting the child; at this juncture the patient thrusts her fingers into her fauces to produce nausea or vomiting; this causes spasmodic, expulsive action in the uterus resulting *not unfrequently* in the immediate birth of the placenta and its membranes; (if such is not the result, there is more or less anxiety; the woman retains her erect position and is *"lomied"* over the womb and abdomen, (a sort of kneading, squeezing operation generally performed with the hands of the attendant,) until the "flowing" is moderate or almost ceases; she is then conducted to a stream, or large container, of water where she is washed down *secundum artem* and re-dressed to return to the house and its promiscuous inhabitants, children and all being often allowed to witness the performance, and think no more of it than they would if it were occurring among the ordinary animal kingdom; this is partly owing to the fact that the common native thatched house has but one room; curtains are used to form their departments and these are not always "kapu" (taboo).

The water there is never at a very low temperature and this bathing process is not usually injurious to these amphibious beings, and only in one instance have I known a serious result follow the erect posture and practice described above. After returning to her "hikie," or bed, the loming process is resumed and kept up for half an hour or more until the pain ceased and the womb is reduced to nearly its normal size. If "after-pains" come on we give orange leaf tea almost ad libitum, (this did not originate with the Hawaiians.) They repeat the loming daily and several times a day where there is pain or enlargement of the uterus without inflammation. When milk begins to secrete, if there is hardness or pain, each gland is lomied until the hardness and pain nearly or entirely passes away; this softens down the parts so that the child can more easily perform its duty; and there is very little danger of sore nipples and "broken breasts" where this practice is properly and thoroughly performed. Usually no aperient is taken and no restriction as to diet adopted. The child is often during the first two days of its postparturient life given to a "wet nurse," to obtain its nourishment particularly if it has spells of crying.—I ought to have said earlier that the mother is required to drink from a pint to a quart of cold water, as soon as the "kaiewe" (the placenta,) is removed, in order *to wash away all that is deleterious.*

Soon after I went to the Islands I was called to a semi-chiefish woman's house just after her delivery to direct the attendants in dressing the child after the most approved American styles; their common way was to wrap the child in "swaddling clothes;" the cloth they formerly used was called "kapa" and was made from the inner bark of two native species of the mulberry. While at the house I noticed three persons in one corner of the room, two of whom were loming the mother of the child, sometimes with the hands, and sometimes with the elbows, and, as it then seemed to me, very unmercifully.

The next day as I was walking in the street I was accosted by a Hawaiian lady with "*aloha kauka*," (which means love or affectionate greeting to the doctor;) her face was familiar, and, on inquiry, I found that she was the, identical person, who had given birth to the child the day before and whom I had last seen passing through a process which would prodigiously frighten American Obstetricians and their lady patients. Most of the native women are up after delivery and about their work as early as the person above mentioned. They, (the natives,) are very careful as to the length of the "piko," (the cord, which remains attached to the child,) and are always anxious to have it left from six to eight inches long; this is uncovered and allowed to dry up from exposure to the air as much as possible, thus being less repulsive in point of odor than such things usually are with us. When it separates, they, or many of them at least, consider it necessary to deposite it in a place which they call a "pana," (usually this is a creviced rock, ship, or cocoa nut tree,) where it is securely fastened or wedged in with small stones or other materials; and, *mirabile dictu,* "if this is not done the child will surely be a thief."—They have a great repugnance to casting the "after-birth" into a vault, or burying it in the ground; with them it must be burned to ashes; the reason of their so-doing I have never been able to learn; it is probably the result of some superstition. In this connection I might say they never, or seldom ever, bury their dead, but put them into a cave, or building thatched for the purpose. The kings' and chief's bones were cleaned from the flesh and preserved, or deposited, one or more of them, in the volcanic lake of fire and (if I mistake not,) the flesh was burned in a common fire.

My obstetrical practice outside of our foreign community has been principally confined to difficult cases; only in one instance in my practice have I used the forceps and then one blade of it sufficed; in another I attempted the use of them, but gave them up on account of the extreme tenderness of the parts; in a third case where the patient imagining that she felt the instruments in my pocket, and of which she had a perfect horror, jumped up in terror and in doing so pushed me over without thinking that she was treating me uncivilly and would not allow me to render any further assistance until she was convinced that I had with me only an ordinary pocket case of instruments and my usual case of medicines;—fluid extract of ergot, (the form I usually use there,) aided nature in completing the delivery; when this is effected the foreign accoucher's services are generally no longer needed, unless metritis supervenes as it sometimes does, when ordinarily, hot turpentine stupes, followed by flannel, bags of hops, with linseed-meal and spices dipped in hot brandy, applied assiduously to the abdomen, with injections of water as hot as they will bear per vaginam, together with opium internally, effect cures as frequently as they do in America.

Turning I have practiced successfully, but not frequently, believing as I do, that *Blundell's oft repeated maxim is*, in the great majority of instances, *true* the world over, viz: "*Meddlesome midwifery is bad.*"

